# Simultaneous spectrofluorimetic determination of remdesivir and simeprevir in human plasma

**DOI:** 10.1038/s41598-022-26559-3

**Published:** 2022-12-20

**Authors:** Mona E. El Sharkasy, Manar M. Tolba, Fathalla Belal, Mohamed I. Walash, Rasha Aboshabana

**Affiliations:** Department of Pharmaceutical Analytical Chemistry, Faculty of Pharmacy, Mansoura University, Mansoura, 35516 Egypt

**Keywords:** Analytical chemistry, Green chemistry

## Abstract

As new infectious mutations of SARS-CoV-2 emerged throughout the world, innovative therapies to counter the virus-altered drug sensitivities were urgently needed. Several antiviral options have been in clinical trials or in compassionate use for the treatment of SARS-CoV-2 infections in an attempt to minimize both clinical severity and viral shedding. Recent research indicated that simeprevir acts synergistically with remdesivir, allowing for a multiple-fold decrease in its effective dose when used at physiologically acceptable concentrations. The goal of this work is to develop a sensitive synchronous spectrofluorimetric approach to simultaneously quantify the two drugs in biological fluids. Using this method, remdesivir and simeprevir could be measured spectrofluorimetrically at 283 and 341 nm, respectively, without interference from each other using Δλ of 90 nm. The effect of various experimental parameters on the fluorescence intensity of the two drugs was extensively explored and optimized. For each of remdesivir and simeprevir, the method exhibited a linearity range of 0.10–1.10 μg/mL, with lower detection limits of 0.01 and 0.02 μg/mL and quantification limits of 0.03 and 0.05 μg/mL, respectively. The high sensitivity of the developed method permitted the simultaneous determination of both drugs in spiked plasma samples with % recoveries ranging from 95.0 to 103.25 with acceptable standard deviation values of 1.92 and 3.04 for remdesivir and simeprevir, respectively. The validation of the approach was approved by the International Council of Harmonization (ICH) guidelines.

## Introduction

Severe acute respiratory syndrome Coronavirus 2 (SARS-CoV-2) created a pandemic in the last three years with a significant number of fatalities. The disastrous effects prompted a worldwide search for a viable treatment for the causative virus. Despite the fact that various types of vaccines have lately been licensed and released, people's anxiety about immunizations is widespread^1^. The appearance of mutant strains capable of compromising the preventive role of vaccinations seems to be the most recent problem. As a result, and despite the emergence of vaccinations, effective anti-SARS-CoV-2 medication is still urgently required^2^. In the absence of a particular antiviral medication against SARS-CoV-2, as well as the lengthy licensing procedure for a new medicine, experiments were conducted to assess the efficacy of available antiviral agents against the aggressive disease for post-infection therapy.

**Figure 1:**
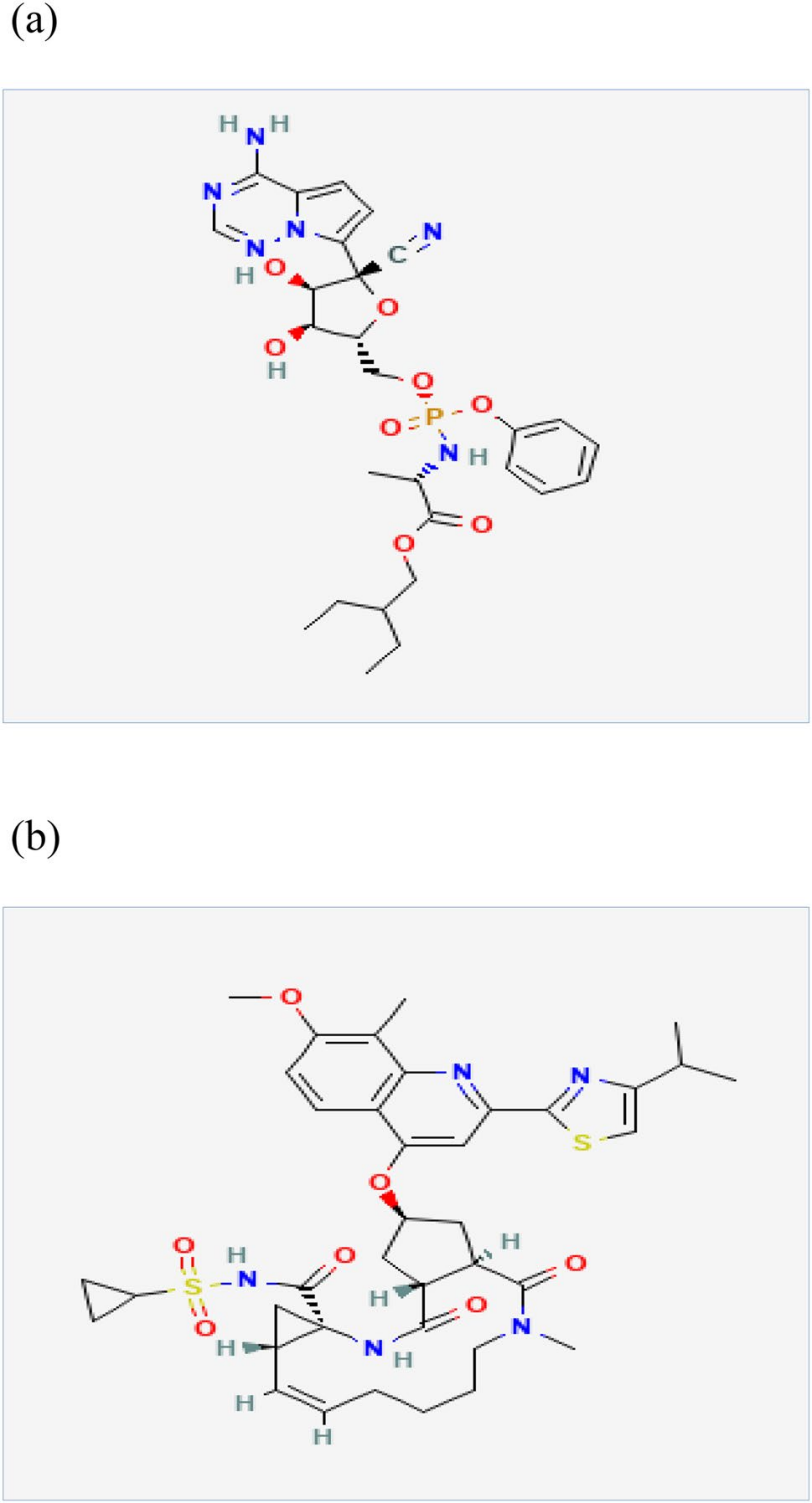
Chemical structure of (**a**) Remdesivir and (**b**) Simeprevir.

Remdesivir (REM, Fig. 1a) is a 1-cyano-substituted nucleotide analogue monophosphoramidate pro-drug. It is first hydrolyzed and then phosphorylated to generate nucleoside triphosphate, which obstructs viral RNA polymerase and evades viral exonuclease proofreading, resulting in a reduction in viral RNA synthesis. It is integrated into nascent viral RNA chains, causing the virus to be terminated prematurely^3–6^. REM has recently gained interest as a possible SARS-CoV-2 medicinal treatment after it has shown significant selectivity and efficacy against the Ebola virus^7^. The literature review indicated that few methods had been reported for the analysis of REM including spectrophotometry^8^, spectrofluorimetry^9^, TLC^10^, and LC^11–18^.

Even though REM is widely recognized as one of the potential therapeutic options for COVID-19 disease, recent randomized clinical trials have only found a minor reduction in disease duration in patients using it. As a result, additional research is needed to identify more effective, easily re-purposed therapeutic medicines for SARS-CoV-2 infections, either as a standalone treatment or in combination with other treatments to improve its efficacy^19^.

Simeprevir (SIM, Fig. 1b), on the other hand, is a second-generation protease inhibitor that has been authorized to treat hepatitis C virus (HCV) infections. The anti-HCV effect of SIM is achieved by suppression of the viral NS3/4A protease, which prevents viral maturation by interfering with protein synthesis^20^. In-vitro, SIM decreases SARS-CoV-2 viral load and inhibits not only the main protease (M pro) but also the RNA-dependent RNA polymerase (RdRp), as well as modulating host immunological responses^19,21^. Interestingly, SIM and REM work synergistically, allowing the effective dose of both to be reduced^19^. The unexpected anti-SARS-CoV-2 mechanism of SIM might contribute to future antiviral strategies. Also, this combination therapy may also assist in reducing the quantity of medication needed to alleviate harmful effects^19^. For the analysis of SIM, the literature review reveals several analytical methods for its assay, whether alone or in combination. These methods involve spectrophotometric^22,23^, chemometric-assisted spectrophotometric^24^, spectrofluorimetric^25–27^, and LC methods^28–33^.

Currently, no method has been reported for simultaneously analyzing REM and SIM in biological fluids as co-administered medications. This means that hospitalized in-patients need a way to measure both medications at the same time so that their therapeutic drug monitoring can be evaluated.

When scanning each REM and SIM on the classical spectrofluorimetric approach, it was noticed that their two emission spectra were intensively overlaid preventing this approach from being used for resolution. Using a constant wavelength synchronous spectrofluorimetric (SFS) method, the problem of spectral overlap was handled.

Although spectrofluorimetric technology is known for its great sensing properties, selectivity issues might occur when studying multi-component combinations due to the overlay of broadband spectra. This overlaying difficulty might be confronted by the employing synchronous technique, as it has several of benefits over standard fluorescence techniques, which include high selectivity, proper spectra, and minimum intrusion^34–36^. SFS is highly basic but proved to be an effective approach for the quantitative assessment of the two drugs in an appropriate time frame because of its narrow and sharp spectra^37–40^. REM and SIM emit separable synchronous fluorescence emission peaks with intensities measurable at 283 nm and 341 nm, respectively, after adopting the constant wavelength synchronous spectrofluorimetric (Δλ) method.

Centered on the pharmacological and chemical characteristics of the aforementioned medications and their enhanced therapeutic efficacy in the management of COVID-19 illness and for the goal of therapeutic medication monitoring, the SFS methodology was exploited for the concomitant quantification of REM and SIM in spiked plasma samples, and the results were satisfactory.

## Experimental

### Instruments and material

#### Instrumentation

- An Agilent G8900A Cary Eclipse Spectrofluorimeter fitted with a Xenon flash lamp was used for the study (Agilent, California, USA). The synchronous method was set using Δ λ = 90 nm, slit width of 5 nm, and factor 20 was utilized to smooth out the obtained spectra. The voltage was 800 V, and the scanning rate was 600 nm/min in the wavelength range of 200–600 nm.
- Microsoft Excel (2010) was used for creating figures and graphs from the data stored in the ASCII. format.
- The pH meter from Consort NV P-901 Belgium was used to adjust the pH of the buffer solutions used.
- The biological samples were mixed with a Vortex IVM-300p from Gemmy Industrial Corporation in Taiwan, then separated with a centrifugal force from Germany's version 2-16P.

#### Materials, chemicals, and reagents

- The raw material of remdesivir was generously donated by EVA Pharma. Co., Cairo, Egypt.
- The raw material of simeprevir sodium was kindly given by AUG Pharma (2nd Industrial Zone, 6 October City, Egypt).
- HPLC grade organic solvents were obtained from a Fisher Scientific distributor in Cairo, Egypt.
- Different surfactants were purchased from Sigma-Aldrich, Germany.
  - El-Nasr Pharmaceutical Chemicals Co., Cairo, Egypt, supplied all other chemicals: acetic acid (96%), phosphoric acid, boric acid, and sodium hydroxide.
  - The plasma sample was collected from Mansoura University Hospital's National Egyptian Blood Bank, Mansoura, Egypt. After mild thawing, it was kept frozen at – 20 °C until required.

#### Standard solutions

In a 50 mL volumetric flask, 0.01 gm of each of REM and SIM was dissolved in methanol to prepare stock solutions of concentration (200 μg/mL). For subsequent use, the stock solutions were kept refrigerated at 4 °C.

#### Working solutions

Individual working solutions (10.0 μg/mL) of each of REM and SIM were generated individually by transferring 1.25 mL of receptive standard solutions (200 μg/mL) to 25 mL calibrated flasks, then terminating with methanol to the calibrated volume and refrigerating at 4 °C for later use.

#### Preparation of Britton Robinson buffer solutions

A solution of 0.04 M of each of acetic acid, boric acid, and phosphoric acid was used to generate Britton Robinson buffer (BRb) solutions. With 0.2 M sodium hydroxide, the pH of BRb solutions was changed to create several solutions within pH ranges of 2.1–12.0.

### Procedures

#### Construction of calibration graphs

Aliquots of REM and SIM standard solutions within the working concentration range listed in Table 1 were transferred into a set of 10 mL volumetric flasks, diluted to volume with ethanol, and mixed well. The synchronous fluorescence spectra of the prepared solutions were measured at a wavelength difference of 90 nm. The fluorescence intensities were recorded at 283 nm and 341 nm for REM and SIM, respectively. At the same time, a blank solution was carried out. The calibration plots were then created by plotting relative synchronous fluorescence intensity (RSFI) versus ultimate drug concentrations, and then the relevant regression equations were derived.

#### Analytical procedure for REM/SIM in lab-prepared mixtures

To verify the method's specificity, standard solutions encompassing multiple proportions of REM and SIM were diluted in 10 mL volumetric flasks with ethanol to reach the appropriate concentration. The nominal content was determined using its corresponding regression equation.

#### REM/SIM analysis in spiked human plasma

This study has been carried out to analyze REM and SIM in spiking human plasma at the same time in order to determine their pharmacological concentration. The same volume of plasma sample (1.0 mL) was transferred into a set of screw-capped centrifugal tubes with a capacity of 13.0 mL and mixed individually with varying amounts of REM and SIM working solutions and vortex-mixed for 60 s. The protein was then precipitated by adding up to 5 mL of acetonitrile to the mixture. The screw-capped tubes were vortex mixed for 30 s before centrifugation at 3600 rpm for 30 min. Then, 1.0 mL aliquots of the upper clear layer were withdrawn, filtered through 0.45 μm syringe filters, transferred to volumetric flasks (10.0 mL), and diluted with ethanol to the labeled volume. A blank solution was treated and measured in parallel, and the assessment was carried out as mentioned under “Construction of calibration graphs”. Using the appropriate regression analysis, the nominal plasma content was estimated.

### Ethical approval

No human subjects or experimental animals were involved in this study. No real plasma samples were included in this study. Plasma sample was "pooled plasma", obtained from the National Egyptian Blood Bank at Mansoura University Hospital, Mansoura, Egypt.

## Results and discussion

**Figure 2:**
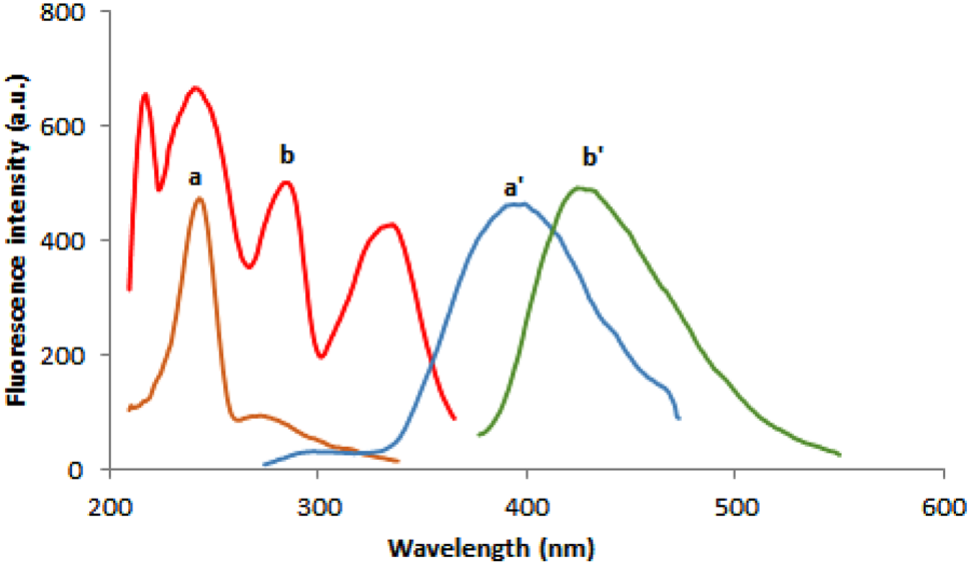
Excitation and emission spectra of 0.05 μg/mL REM (a, a′) and 0.5 μg/mL SIM (b, b′) in ethanol.

**Figure 3:**
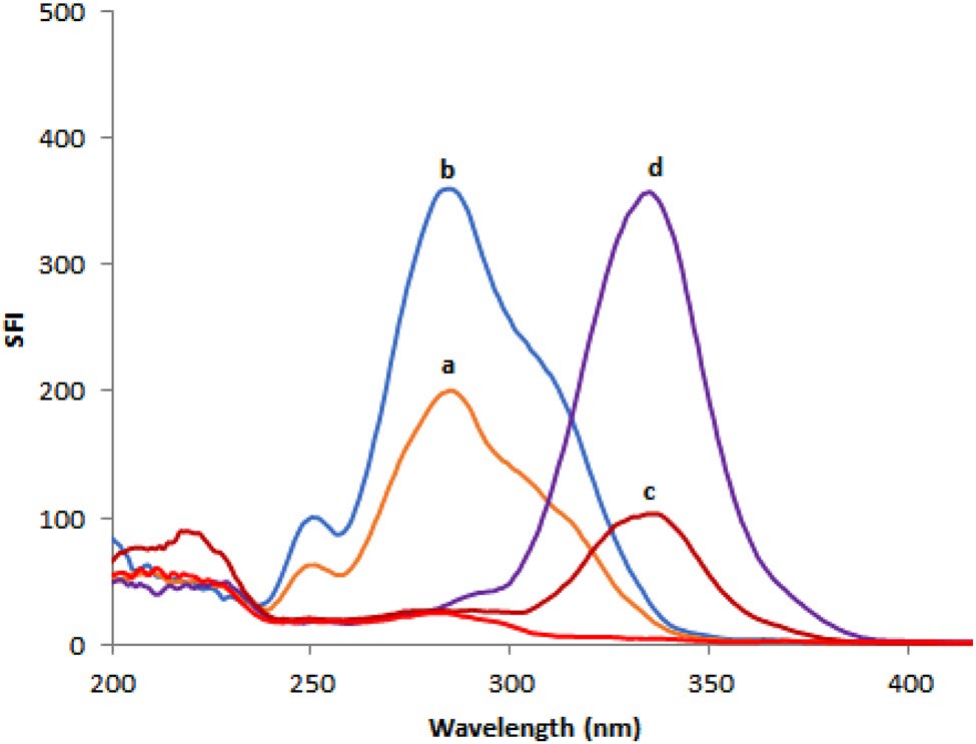
Synchronous fluorescence spectra indicating resolved peaks of the studied drugs. Where: (a, b): 0.2 and 0.4 μg/mL REM, (c, d): 0.1 and 0.4 μg/mL SIM in ethanol (blank solvent).

**Figure 4:**
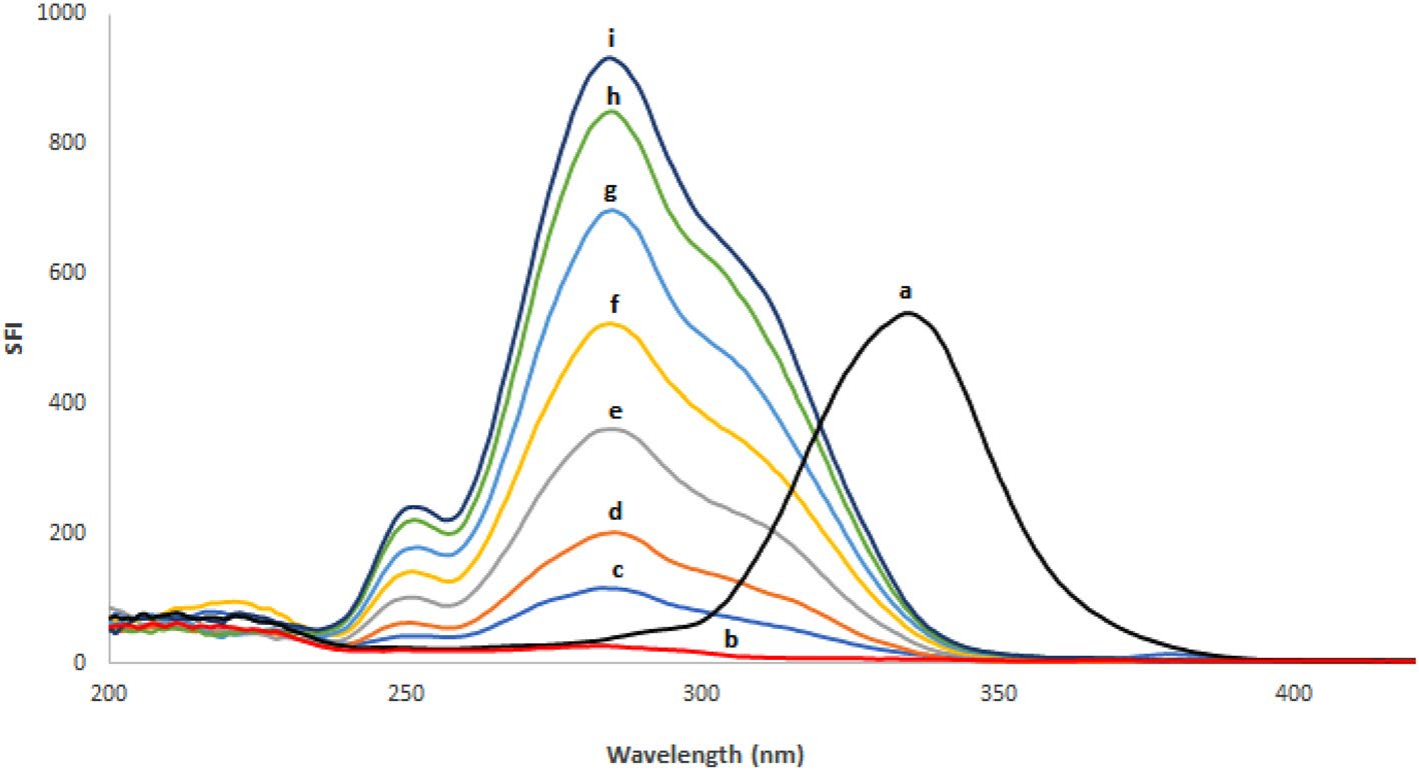
Synchronous fluorescence spectra of (a) SIM (0.60 μg/mL), (b) Blank and (c-i: 0.10, 0.20, 0.40, 0.60, 0.80, 1.00, 1.10 μg/mL) of REM at Δλ = 90 nm.

**Figure 5:**
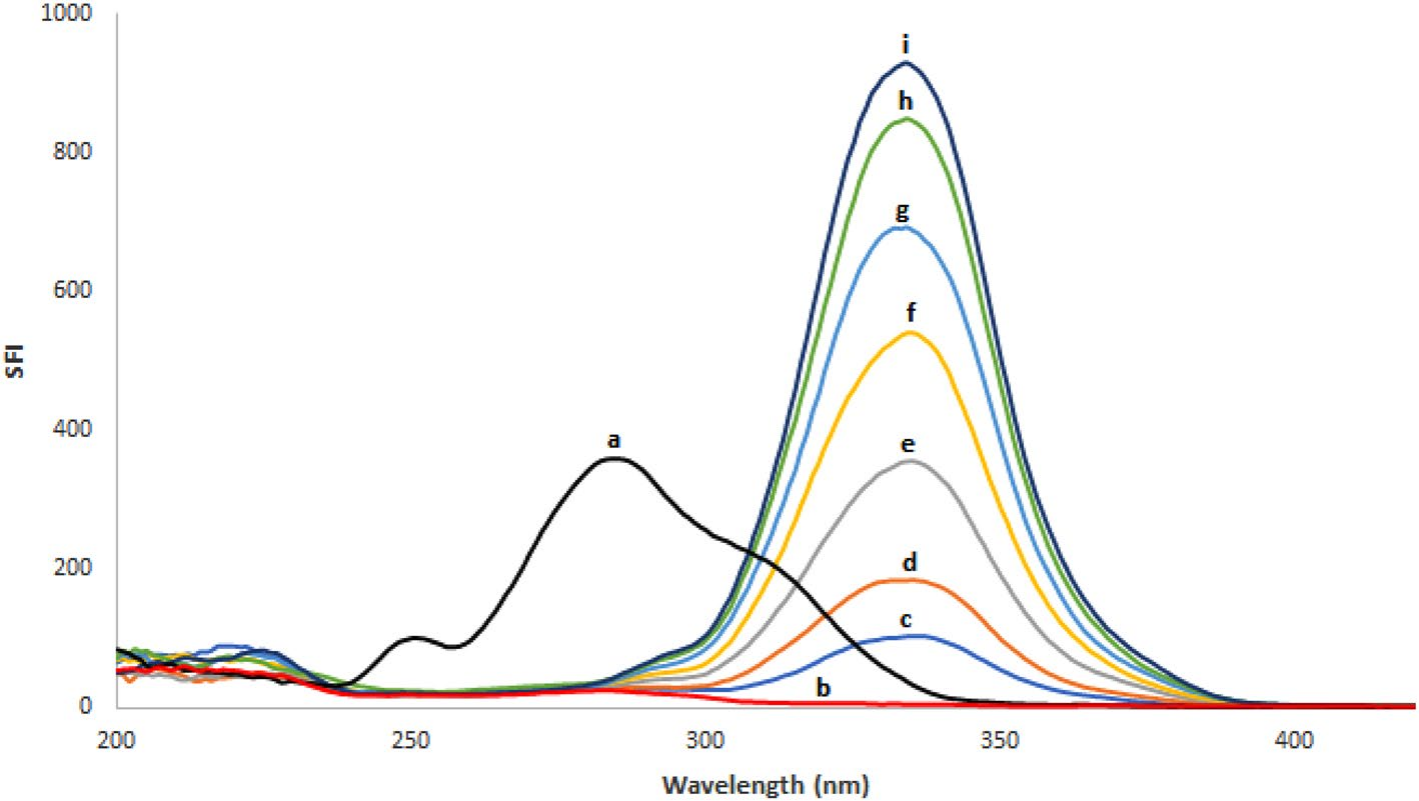
Synchronous fluorescence spectra of (a) REM (0.40 μg/mL), (b) Blank and (c-i: 0.10, 0.20, 0.40, 0.60, 0.80, 1.00, 1.10 μg/mL) of SIM at Δλ = 90 nm.

Based on the relevance of SFS in pharmaceutical analysis, a sensitive and simple spectrofluorimetric approach for measuring REM and SIM in spiked plasma samples was created. As shown in Fig. 2, both ethanolic solutions of REM and SIM emit fluorescence at wavelengths of 400 and 429 nm upon excitation at 243 and 284 nm, respectively. Detailed examination of Fig. 2 reveals that the REM and SIM emission spectra are significantly overlapped, rendering simultaneous analysis of such a combination a challenging task. As a result, it was recommended to use the extremely sensitive and selective SFS approach to enhance its selectivity. Typical SF spectra for both REM and SIM were reported at Δλ of 90 nm, and it was found that their peaks were well-defined, allowing each drug to be analyzed without interference from the other. The synchronized fluorescence intensity of REM and SIM could be measured with high sensitivity at 283 nm and 341 nm, respectively (Fig. 3). Different concentrations of REM were measured at 283 nm in the presence of a fixed concentration of SIM (0.60 μg/mL) as shown in Fig. 4, whereas SIM was detected at 341 nm in the presence of a fixed concentration of REM (0.40 μg/mL) as shown in Fig. 5. All of the fluorescence measurements were obtained at Δλ of 90 nm.

### Optimization of experimental factors

The effect of various investigational conditions on the fluorescence intensities of the medications described above was explored and optimized. Each variable was evaluated independently, while the others remained unchanged. The value of Δλ was found to deeply affect the SF scanning process. It also has a substantial impact on sensitivity as well as peak resolution. As shown in Fig. S1, both REM and SIM were scanned at various Δλ values ranging from 20 to 160 nm. The SF spectra of the indicated drugs were produced in a single step with reasonable sensitivity using Δλ value of 90 nm, which proved to be appropriate as it gave a clear response for both substances. Fluorescence intensities for both medications were reduced when Δλ was lowered below 90 nm. At Δλ above 90 nm, both medications fluoresced intensely but with overlapped spectra. The optimal conditions for fluorescence signal and segregation of the two overlapped spectra were determined using a diversity of solvent solutions, including distilled water, acetone, acetonitrile, methanol, and ethanol. Both drugs were scanned at Δλ = 90 nm in different solvents and compared in term of sensitivity as well as spectral resolution. When it came to REM, ethanol had the greatest fluorescence values, followed by acetonitrile and methanol, respectively, while water had the lowest fluorescence signal of all the diluents tested. When it comes to SIM, distilled water generated relatively low fluorescence readings related to the organic solvents, including; ethanol, acetonitrile, and methanol. Acetone also significantly reduced the SFIof both drugs. Because of this, ethanol was adopted in this study as the principal diluent owing to sensitivity and environmental concerns, as shown in Fig. S2. Britton Robinson buffer solutions with a pH range of 2.1–12.0 were employed in this investigation. It was noticed that changes in pH had no effect on the SFI of both drugs over the whole pH range. This result matches with literature^9^ in which REM was determined utilizing its native fluorescence at 244/405 nm. It was reported that REM fluorescence intensity was studied using the BRb buffer and exhibited nearly similar intensities within the pH range of 3.0–5.0 and such intensity started to decrease above pH 6.0. As a result, no buffer solutions were used in the suggested method. Different organized media were studied, using non-ionic surfactant (Tween-80), anionic surfactant (SDS), anionic polysaccharide (CMC), macromolecules (β-CD) and cationic surfactant (cetrimide). All are prepared in distilled water at concentrations greater than the critical micelle level and tested to see if they can improve fluorescence readings of the aforementioned drugs^41^. As shown in Fig. S3, neither surfactants nor macromolecules resulted in a considerable increase in the fluorescence emission of REM when compared to ethanol as the selected green diluent. This observation may be explained by the bulky nature of the drug and consequently the steric hindrance preventing its inclusion in the cavities of the micelles. While for SIM, only tween-80 produces a significant increase in fluorescence signal compared to other surfactants and macromolecule. This signal is nearly equal to that of ethanol. As a result, in the proposed method, ethanol was chosen as the best solvent for both drugs without the use of a buffer or organized media.

### Method validation

To determine whether the proposed approach was valid or not, the International Conference on Harmonization (ICH) Q2 (R1) guidelines were adopted^42^.

#### Range and linearity

For both medications, a linear correlation was established between the values of relative synchronous fluorescence intensity (RSFI) and the ultimate drug concentrations across the range of 0.10–1.10 μg/mL. Table 1 presents a summary of the obtained ranges, regression equations, and calibration curve data. The calibration curves' linearity was confirmed by statistical analysis of the data^43^ and values approaching the unity of correlation coefficients.

**Table 1:** Analytical performance data for the proposed method.

Validation parameter	REM	SIM
Wavelength difference (Δλ)	90 nm	
Linearity range (μg/mL)	0.10–1.10	0.10–1.10
Intercept (a)	11.80	15.17
Slope (b)	802	747
Correlation coefficient (r)	0.9999	0.9999
S.D. of residuals (S_y/x_)	3.15	5.12
S.D. of intercept (S_a_)	2.32	3.77
S.D. of slope (S_b_)	3.32	5.40
Limit of detection, LOD (μg/mL)	0.01	0.02
Limit of quantitation, LOQ (μg/mL)	0.03	0.05

#### Limit of quantitation (LOQ) and limit of detection (LOD)

LOQ and LOD values are depicted in Table 1. The acquired values demonstrated that the suggested approach can accurately quantify the previously stated medications in spiked plasma and, hence, may be used in future in vivo research.

#### Accuracy and precision

The suggested method's accuracy was confirmed by statistically analyzing the obtained data compared to those obtained using reference methods^9,25^. The Student t-test and the Variance ratio F-test were used to statistically examine the data^43^. The results in Table 2 reveal that there was no substantial difference in performance between them in terms of precision and accuracy.

**Table 2:** Application of the proposed method for the determination of the studied drugs in their raw materials.

Parameters	REM			SIM		
	Amount taken (μg/mL)	Amount found (μg/mL)	% found^a^	Amount taken (μg/mL)	Amount found (μg/mL)	% found^a^
	0.10	0.099	99.00	0.10	0.099	99.00
	0.20	0.200	100.00	0.20	0.199	99.50
	0.40	0.399	99.75	0.40	0.403	100.75
	0.60	0.599	99.83	0.60	0.593	98.83
	0.80	0.807	100.88	0.80	0.811	101.38
	1.00	0.996	99.60	1.00	1.001	100.10
	1.10	1.099	99.91	1.10	1.094	99.45
Mean ± SD			99.85 ± 0.56			99.86 ± 0.94
% RSD			0.56			0.94
% Error			0.21			0.35

	Comparison method (n = 3)^9^	Comparison method (n = 3)^25^				
Mean ± SD	99.98 ± 0.66	100.02 ± 0.97				
t^b^	0.26 (2.31)^b^	0.24 (2.31)^b^				
F^b^	1.39 (5.14)^b^	1.06 (5.14)^b^				

^a^Average of 3 replicate determinations.

^b^The values between parentheses are the tabulated values of *t* and *F* at *P* = 0.05^43^.

The precision of the developed approach was evaluated to determine inter-day and intra-day precisions. The medications under study had low percent RSD and percent error, indicating that the established procedures are sufficiently precise (Table 3).

**Table 3:** Inter-day and intra-day precision data for the studied drugs using the proposed method.

Concentration (μg/mL)	Intra-day precision			Inter-day precision		
	Mean ± SD	% RSD	% error	Mean ± SD	% RSD	% error
**REM**						
0.20	101.01 ± 0.93	0.92	0.53	100.57 ± 0.79	0.79	0.46
0.40	100.34 ± 1.07	1.07	0.62	100.88 ± 0.92	0.91	0.53
0.80	100.65 ± 1.13	1.12	0.65	99.86 ± 1.04	1.04	0.60
**SIM**						
0.10	99.40 ± 1.44	1.45	0.84	99.89 ± 1.47	1.47	0.85
0.40	98.98 ± 1.40	1.41	0.81	99.85 ± 1.53	1.53	0.88
0.80	101.13 ± 0.51	0.50	0.29	100.20 ± 0.90	0.90	0.52

#### Selectivity

The anticipated methodology was utilized to analyze REM and SIM at the same time with good recoveries and without interference, as cited in Table 4. The recommended approach's selectivity was further proven by quantifying the cited drugs in complicated human plasma matrices. The procedure was demonstrated to have adequate selectivity to examine the previously stated drugs with high percent recoveries (95.0–103.25%) and low percent RSD values, pointing out that there was no disruption from plasma intrinsic constituents.

**Table 4:** Assay results for the determination of REM and SIM in synthetic mixtures using the proposed method.

Mix no.	Amount taken (μg/mL)	Amount taken (μg/mL)	Amount found (μg/mL)	Amount found (μg/mL)	% found^a^	% found^a^
	REM	SIM	REM	SIM	REM	SIM
1	0.40	0.60	0.403	0.600	100.75	100.00
2	0.50	0.50	0.496	0.502	99.20	100.40
3	0.20	0.80	0.199	0.809	99.50	101.13
4	1.00	0.50	1.002	0.495	100.20	99.00
5	0.30	0.60	0.305	0.595	101.67	99.17
Mean					100.26	99.94
± SD					0.99	0.88
% RSD					0.99	0.88

^a^Mean of three determinations.

### Applications of the suggested procedure

#### REM/SIM analysis in lab-prepared mixtures

**Figure 6:**
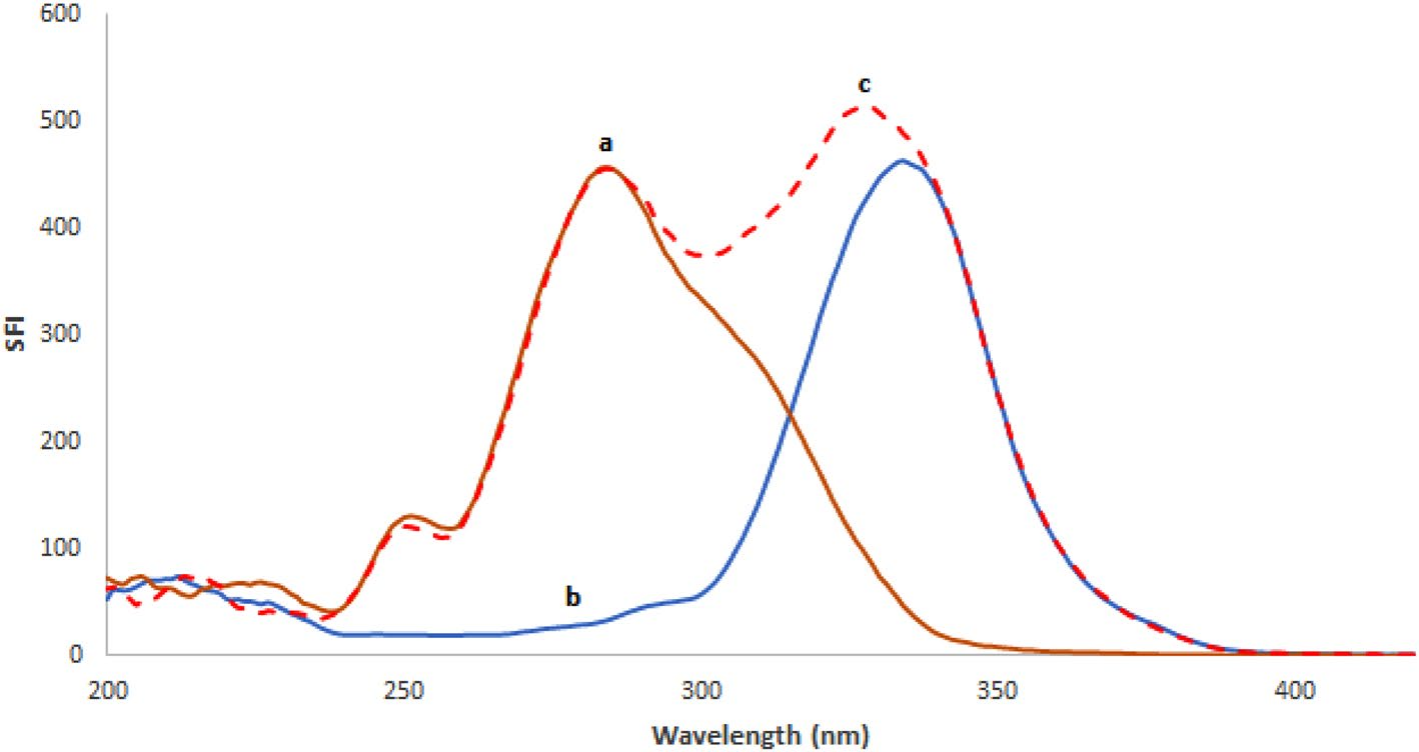
Synchronous fluorescence spectra of: (a) 0.50 μg/mL REM, (b) 0.50 μg/mL SIM, and (c) synthetic mixture of both.

As shown in Fig. 6, the indicated process was utilized to analyze synthetic combinations with varied REM and SIM proportions. The amounts of the specified medications in their synthetic mixtures might be calculated using the corresponding regression equations. The obtained results, as shown in Table 4, revealed the accuracy of the designated procedure.

#### Analysis of human plasma samples spiked with REM and SIM

**Figure 7:**
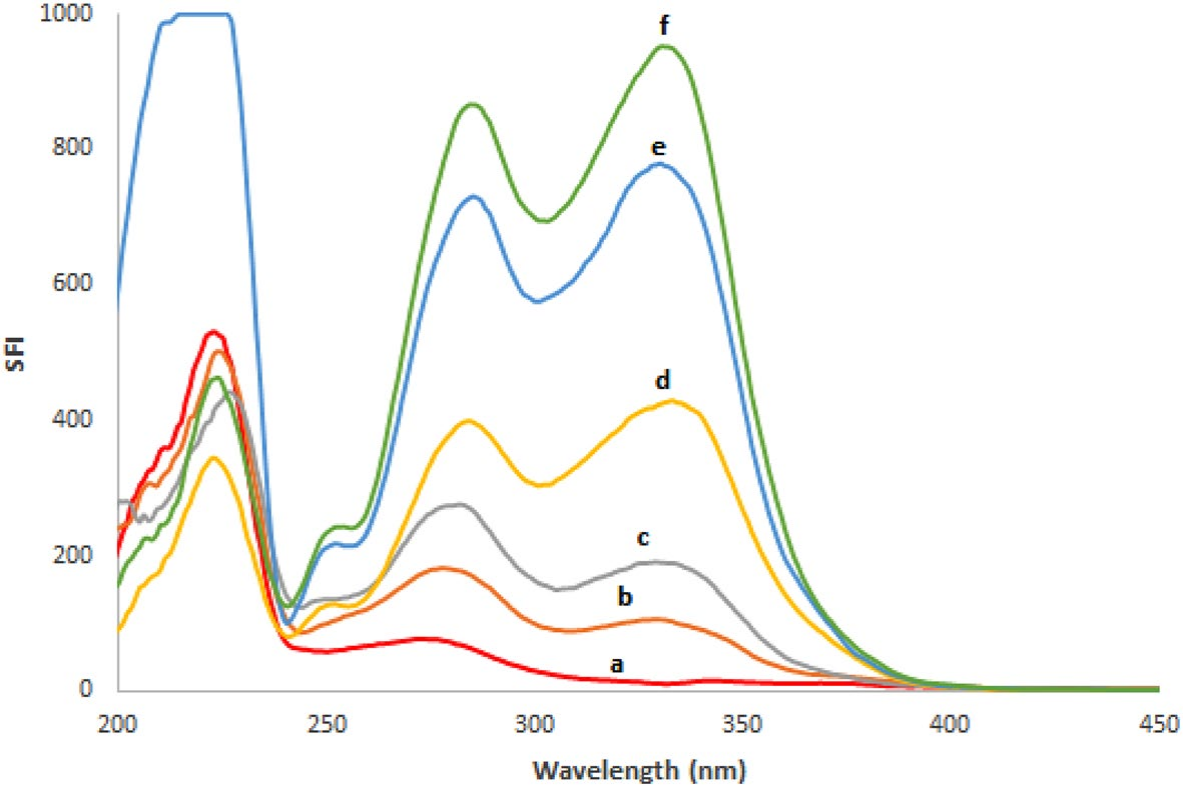
Application of drugs in spiked human plasma where: (a) Blank (Plasma). (b–f) concentration of drugs spiked in plasma samples (0.10, 0.20, 0.40, 0.80, 1.00 μg/mL) for each of REM and SIM, respectively.

For the treatment of Covid-19, Gilead recommends a 200 mg loading intravenous dose of REM on the first day followed by nine days of 100 mg once daily to target viral exposures in plasma and cells^44^. It was documented that C_max_ of REM on the first day was 5.44 μg/mL and on the fifth day, it was 2.61 μg/mL^44^. While C_max_ of SIM was reported to be approximately 2.588 μg/mL after multiple 150 mg once-daily oral doses, with a t_max_ of 4–9 h^45^. The high sensitivity of the developed techniques down to 0.01 and 0.02 μg/mL for REM and SIM, respectively- enabled for simultaneous assessment of the two medicines in biofluids. A linear association was demonstrated when the RSFI of REM and SIM in spiked samples were plotted against the ultimate drug concentrations (μg/mL) as illustrated in Table 5. The devised approach had acceptable percent recoveries (95.0–103.25%) and low percent RSD values of 1.92 and 3.04 for REM and SIM, respectively, implying that it might be used for in-patient therapeutic drug monitoring of the specified drugs as illustrated in Fig. 7.

**Table 5:** Application of the proposed spectrofluorimetric method for the determination of the studied drugs in spiked human plasma.

Parameters	REM			SIM		
	Amount taken (μg/mL)	Amount found (μg/mL)	% recovery	Amount taken (μg/mL)	Amount found (μg/mL)	% recovery
	0.10	0.098	98.00	0.10	0.099	99.00
	0.20	0.204	102.00	0.20	0.190	95.00
	0.40	0.393	98.25	0.40	0.413	103.25
	0.80	0.814	101.75	0.80	0.808	101.00
	1.00	0.991	99.10	1.00	0.990	99.00
Mean ± SD			99.82 ± 1.92			99.45 ± 3.04
r	0.9997			0.9996		
Regression equation	y = 783.0 x + 30.70			y = 945.5 x + 1.65

### Estimation of the suggested method's greenness

**Figure 8:**
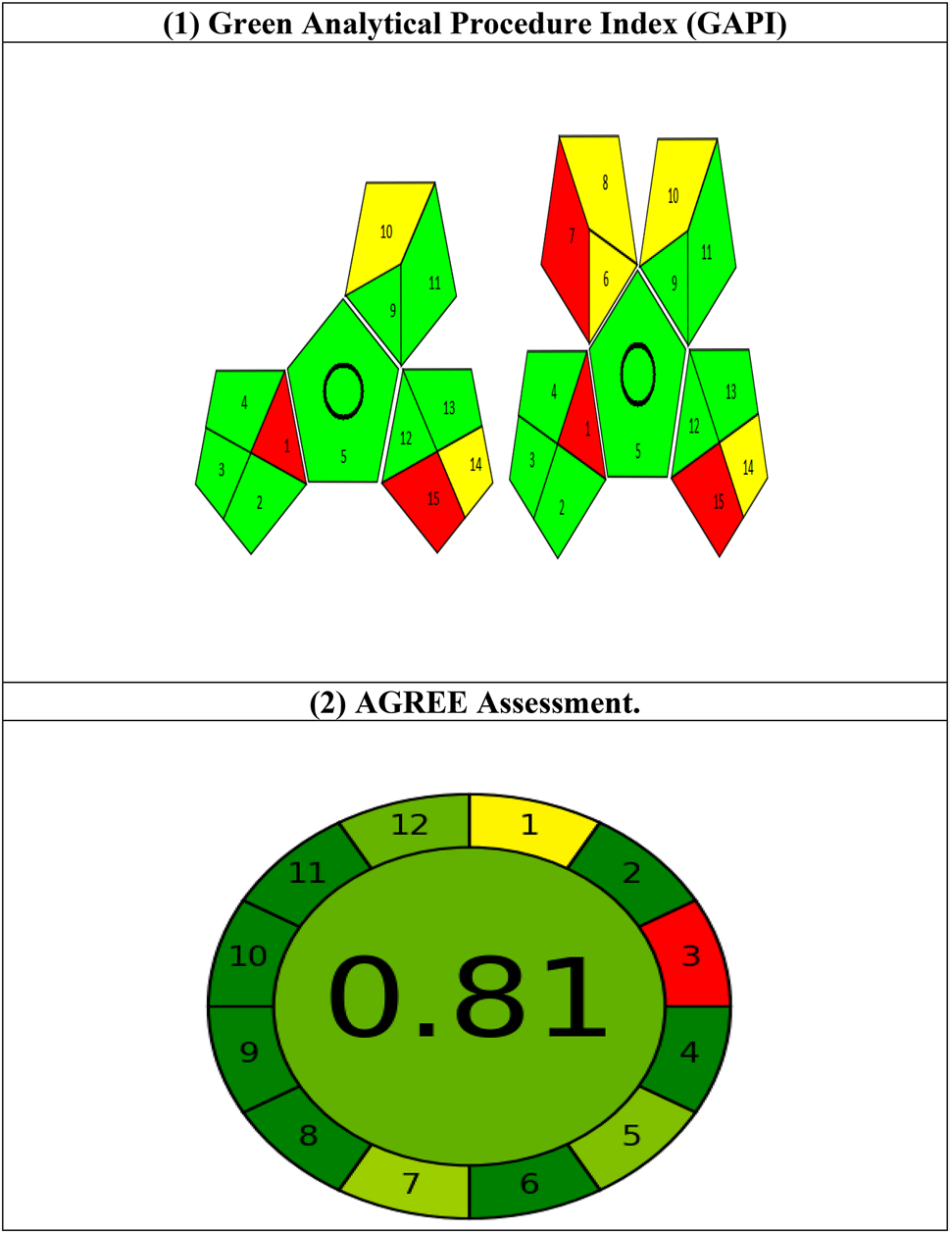
Results for evaluation of the greenness of the developed method by different green analytical chemistry metric tools.

Globally, there is an increased emphasis on managing waste and dangers to enhance green analysis. Analytical GREENNESS metric approach (AGREE)^46^ and Green Assessment Profile Index (GAPI)^47^ are two green metrics that were used in this study. Regarding AGREE, it is a technique for identifying the environmental and occupational dangers involved in the analytical procedure. It provides a clock-shaped chart with a circumference split into 12 pieces, based on the 12 principles of Green Analytical Chemistry^48^. On a color scale, each item is handled as a separate parameter. The center of the AGREE chart indicates the overall acceptance color and assessment score on a scale of 0 to 1 (Fig. 8). A semiquantitative technique called GAPI was also employed for both drugs in raw materials and spiked plasma samples in order to ascertain the green property in each phase. It employs a visual graph to categorize the greenness of each phase in a methodological approach, using a chromaticity scale of red, yellow, and green. The two processes used generate little waste and require only a small amount of non-toxic chemicals. Additionally, the applied procedure was a direct approach that was intended for qualification and quantification. The results, which are adequate and indicate acceptable green approaches, are displayed in Fig. 8.

### Comparison between the proposed method and other reported methods

The proposed method involved the simultaneous determination of a synergistic combination of REM and SIM. The resolution of the overlaid spectra was allowed by using Δλ value of 90 nm, which is not equal to the difference between the emission and excitation wavelengths of the two drugs. Therefore, the sensitivity of the proposed method is slightly lower than the native estimation reported in the literature^9,25^. However, it is higher than another fluorimetric method reported for SIM^25^. As a result, the key advantage of the new spectrofluorimetric technique is energy savings, as the spectrofluorometer requires the least amount of energy (less than 0.1kWh) when compared to HPLC (approximately 1.5kWh) in addition to high sensitivity of the proposed method compared to reported HPLC^12,13,32,33^. Despite the fact that the chromatographic technique is widely employed in pharmaceutical quality control and research laboratories, it is still considered a sophisticated technology due to the high cost of the equipment (column and detector) and the environmental damage caused by the use of hazardous organic solvents. Also, the spectrophotometric techniques are widely used in routine work analysis because of their speed, ease of use, and relative inexpensiveness, but they are deficient in terms of sensitivity^8,23,24^. Additionally, the proposed method is significantly easier, greener (as verified by AGREE and GAPI), cheaper, and time-saving, as proven in (Table 6).

**Table 6:** Comparison between the proposed spectrofluorimetric method and other reported methods.

Method	REM				SIM			
	linearity (μg/mL)	LOD (μg/mL)	LOQ (μg/mL)	Ref	Linearity (μg/mL)	LOD (μg/mL)	LOQ (μg/mL)	Ref.
Proposed method	0.1–1.1	0.01	0.03		0.1–1.1	0.02	0.05	
Spectrofluorimetry	0.001–0.065	0.287 × 10^–3^	0.871 × 10^–3^	9	0.2–2.0	0.04	0.13	27
	–				0.05–1.0	0.016	0.048	25
					0.06–1.5	0.009	0.027	26
HPLC/UV	5.0–100.0	0.5	2.0	12	1.0–20.0	0.03	0.09	33
	0.025–2.5	1.95 × 10^–3^	6.49 × 10^–3^	15	–			
HPLC/DAD	0.1–15.0	0.03	0.1	13	1.5–45.0	0.43	1.30	32
HPLC/FD	0.05–15.0	0.015	0.05		0.01–3.0	–	0.01	31
Spectrophotometry	1.0–20.0	0.05	0.16	8	3.0–45.0	0.89	2.69	23
	–				2.5–45.0	0.71	2.16	24

## Conclusion

The determination of the co-administered antiviral drugs in biological fluids represents a very imperative and pertinent step in the treatment of SARS-CoV-2 illness, and this requires therapeutic drug monitoring. This work analyzes remdesivir and simeprevir concurrently without interference from plasma endogenous components using a very green-sensitive synchronous spectrofluorimetric method. The utilized instrument is straightforward and inexpensive compared to other tools, such as HPLC and gas chromatography. The solvent used in the suggested technique is ethanol, which is green, less expensive, and more widely accessible. Because of the environmental benefits of using green solvents, the technique can be regarded as a viable quality control alternative.

## Supplementary Information

Supplementary Figures.

## Data Availability

All the data generated or analysed during this study are included in the Supplementary File.

## References

[1] Vergara RJD, Sarmiento PJD, Lagman JDN (2021). Building public trust: A response to COVID-19 vaccine hesitancy predicament. J. Public Health.

[2] Wibmer CK (2021). SARS-CoV-2 501Y. V2 escapes neutralization by South African COVID-19 donor plasma. Nat. Med..

[3] Siegel, D. *et al.* (ACS Publications, 2017).

[4] Gordon CJ (2020). Remdesivir is a direct-acting antiviral that inhibits RNA-dependent RNA polymerase from severe acute respiratory syndrome coronavirus 2 with high potency. J. Biol. Chem..

[5] Al-Tawfiq JA, Al-Homoud AH, Memish ZA (2020). Remdesivir as a possible therapeutic option for the COVID-19. Travel Med. Infect. Dis..

[6] Wang M (2020). Remdesivir and chloroquine effectively inhibit the recently emerged novel coronavirus (2019-nCoV) in vitro. Cell Res..

[7] Lo MK (2020). Remdesivir targets a structurally analogous region of the Ebola virus and SARS-CoV-2 polymerases. Proc. Natl. Acad. Sci..

[8] Elama HS, Zeid AM, Shaalan SM, El-Sayed YE-S, Eid MI (2022). Eco-friendly spectrophotometric methods for determination of remdesivir and favipiravir; the recently approved antivirals for COVID-19 treatment. Spectrochim. Acta A.

[9] Elmansi H, Ibrahim AE, Mikhail IE, Belal F (2021). Green and sensitive spectrofluorimetric determination of Remdesivir, an FDA approved SARS-CoV-2 candidate antiviral; application in pharmaceutical dosage forms and spiked human plasma. Anal. Methods.

[10] Noureldeen DA, Boushra JM, Lashien AS, Hakiem AFA, Attia TZ (2021). Novel environment friendly TLC-densitometric method for the determination of anti-coronavirus drugs “Remdesivir and Favipiravir”: Green assessment with application to pharmaceutical formulations and human plasma. Microchem. J..

[11] Du P, Wang G, Yang S, Li P, Liu L (2021). Quantitative HPLC-MS/MS determination of Nuc, the active metabolite of remdesivir, and its pharmacokinetics in rat. Anal. Bioanal. Chem..

[12] Ibrahim AE, Deeb SE, Abdelhalim EM, Al-Harrasi A, Sayed RA (2021). Green stability indicating organic solvent-free HPLC determination of remdesivir in substances and pharmaceutical dosage forms. Separations.

[13] Hamdy MM, Abdel Moneim MM, Kamal MF (2021). Accelerated stability study of the ester prodrug remdesivir: Recently FDA-approved Covid-19 antiviral using reversed-phase-HPLC with fluorimetric and diode array detection. Biomed. Chromatogr..

[14] Reddy HR, Pratap S, Chandrasekhar N, Shamshuddin S (2021). A novel liquid chromatographic method for the quantitative determination of degradation products in remdesivir injectable drug product. J. Chromatogr. Sci..

[15] Jitta SR, Salwa Kumar L, Gangurde PK, Verma R (2021). Development and validation of high-performance liquid chromatography method for the quantification of remdesivir in intravenous dosage form. Assay Drug Dev. Technol..

[16] Moneim MMA, Kamal MF, Hamdy MM (2021). Rapid sensitive bioscreening of remdesivir in COVID-19 medication: Selective drug determination in the presence of six co-administered therapeutics. Rev. Anal. Chem..

[17] Habler K (2021). Simultaneous quantification of seven repurposed COVID-19 drugs remdesivir (plus metabolite GS-441524), chloroquine, hydroxychloroquine, lopinavir, ritonavir, favipiravir and azithromycin by a two-dimensional isotope dilution LC–MS/MS method in human serum. J. Pharm. Biomed. Anal..

[18] Bulduk I, Akbel E (2021). A comparative study of HPLC and UV spectrophotometric methods for remdesivir quantification in pharmaceutical formulations. J. Taibah Univ. Sci..

[19] Lo HS (2021). Simeprevir potently suppresses SARS-CoV-2 replication and synergizes with remdesivir. ACS Cent. Sci..

[20] Rosenquist Å (2014). Discovery and development of simeprevir (TMC435), a HCV NS3/4A protease inhibitor. J. Med. Chem..

[21] Bafna K (2020). Hepatitis C Virus drugs simeprevir and grazoprevir synergize with remdesivir to suppress SARS-CoV-2 replication in Cell Culture. BioRxiv.

[22] Mohamed SH, Issa YM, Salim AI (2021). An eco-concerned development of a fast, precise and economical spectrophotometric assay for the antiviral drug simeprevir based on ion-pair formation. Biointerface Res. Appl. Chem..

[23] Ramzy S, Abdelazim AH (2022). Application of different spectrophotometric methods for quantitative analysis of direct acting antiviral drugs simeprevir and sofosbuvir. Spectrochim. Acta A.

[24] Attia KA, El-Abasawi NM, El-Olemy A, Serag A (2018). Different spectrophotometric methods applied for the analysis of simeprevir in the presence of its oxidative degradation product: A comparative study. Spectrochim. Acta A.

[25] Mohammed BS, Hamad AE, El-Malla SF, Derayea SM (2020). Sensitive spectrofluorimetric assay based on micelle enhanced protocol for the determination of hepatitis C antiviral agent (simeprevir): Application to dosage form and human plasma. Microchem. J..

[26] Hamad AE, Mohammed BS, Derayea SM, El-Malla SF (2020). Micelle sensitized synchronous spectrofluorimetric approaches for the simultaneous determination of simeprevir and ledipasvir: Application to pharmaceutical formulations and human plasma. Spectrochim. Acta A.

[27] Attia KA, El-Abasawi NM, El-Olemy A, Serag A (2018). Simeprevir oxidative degradation product: Molecular modeling, in silico toxicity and resolution by synchronous spectrofluorimetry. Luminescence.

[28] Nannetti G (2016). Development of a simple HPLC–UV method for the determination of the hepatitis C virus inhibitor simeprevir in human plasma. J. Pharm. Biomed. Anal..

[29] Vanwelkenhuysen I, De Vries R, Timmerman P, Verhaeghe T (2014). Determination of simeprevir: A novel, hepatitis C protease inhibitor in human plasma by high-performance liquid chromatography–tandem mass spectrometry. J. Chromatogr. B.

[30] Ferrari D (2019). A liquid chromatography-tandem mass spectrometry method for simultaneous determination of simeprevir, daclatasvir, sofosbuvir, and GS-331007 applied to a retrospective clinical pharmacological study. J. Chromatogr. B.

[31] Youssef AF, Issa YM, Nabil KM (2020). Development and validation of a new method for the determination of anti-hepatitis C agent simeprevir in human plasma using HPLC with fluorescence detection. Curr. Anal. Chem..

[32] Attia KA, El-Abasawi NM, El-Olemy A, Serag A (2017). Stability-indicating HPLC-DAD method for the determination of simeprevir. Anal. Chem. Lett..

[33] Ezzeldin E (2020). Validated reversed-phase liquid chromatographic method with gradient elution for simultaneous determination of the antiviral agents: Sofosbuvir, ledipasvir, daclatasvir, and simeprevir in their dosage forms. Molecules.

[34] Lakowicz JR (1992). Topics in Fluorescence Spectroscopy: Principles.

[35] Magdy G, Belal FF, Abdel-Megied AM, Hakiem AFA (2021). Micelle-enhanced conventional and synchronous spectrofluorimetric methods for the simultaneous determination of lesinurad and febuxostat: Application to human plasma. Spectrochim. Acta A.

[36] Magdy G, Belal F, Abdel-Megied AM, Abdel Hakiem AF (2021). Two different synchronous spectrofluorimetric approaches for simultaneous determination of febuxostat and ibuprofen. R. Soc. Open Sci..

[37] Patra D, Mishra A (2002). Recent developments in multi-component synchronous fluorescence scan analysis. TrAC Trends Anal. Chem..

[38] Attia KA (2021). Application of different spectrofluorimetric methods for determination of lesinurad and allopurinol in pharmaceutical preparation and human plasma. Spectrochim. Acta A.

[39] Barghash S, Elmansi H, Abd El-Razeq S, Belal F (2021). Novel spectrofluorimetric technique for determination of amoxicillin and ethopabate in chicken tissues, liver, kidney, eggs, and feed premix. Luminescence.

[40] Elmansi H, Zayed S, Belal F (2020). Rapid fluorometric determination of ticagrelor in tablets and rat plasma: Application to pharmacokinetics study. Spectrochim. Acta A.

[41] Fluksman A, Benny O (2019). A robust method for critical micelle concentration determination using coumarin-6 as a fluorescent probe. Anal. Methods.

[42] ICH Harmonised Tripartite Guidelines. *Validation of Analytical Procedures: Text and Methodology Q2 (R1)*. http://www.ich.org/products/guidelines/quality/article/quality-guidelines.html. Accessed May 2022.

[43] Miller J, Miller JC (2005). Statistics and Chemometrics for Analytical Chemistry.

[44] Agency EM (2020). Summary on compassionate use remdesivir gilead. Eur. Med. Agency.

[45] Bourgeois S (2017). Pharmacokinetic interactions between simeprevir and ledipasvir in treatment-naive hepatitis C virus genotype 1-infected patients without cirrhosis treated with a simeprevir-sofosbuvir-ledipasvir regimen. Antimicrob. Agents Chemother..

[46] Pena-Pereira F, Wojnowski W, Tobiszewski M (2020). AGREE: Analytical GREEnness metric approach and software. Anal. Chem..

[47] Płotka-Wasylka J (2018). A new tool for the evaluation of the analytical procedure: Green analytical procedure index. Talanta.

[48] Gałuszka A, Migaszewski Z, Namieśnik J (2013). The 12 principles of green analytical chemistry and the SIGNIFICANCE mnemonic of green analytical practices. TrAC Trends Anal. Chem..

