# Supplementary material for: Simultaneous spectrofluorimetic determination of remdesivir and simeprevir in human plasma
Source: Sci Rep. 2022 Dec 20;12:21980. doi: 10.1038/s41598-022-26559-3 (PMC9763795; doi:10.1038/s41598-022-26559-3)
Supplement: Supplementary file 1 — Supplementary Figures. [file 41598_2022_26559_MOESM1_ESM.docx]

**Supplementary material figure captions:**

**Fig. S1**: Optimization of delta wavelength (Δλ) for synchronous fluorescence spectra of 0.50 μg/mL for each of REM and SIM.

| **Fig. S2**: Effect of diluting solvent on synchronous fluorescence intensity of 0.50 μg/mL for each of REM and SIM.  **Fig. S3:** Effect of surfactants on synchronous fluorescence intensity of 0.50 μg/mL for each of REM and SIM. |
| --- |
|  |


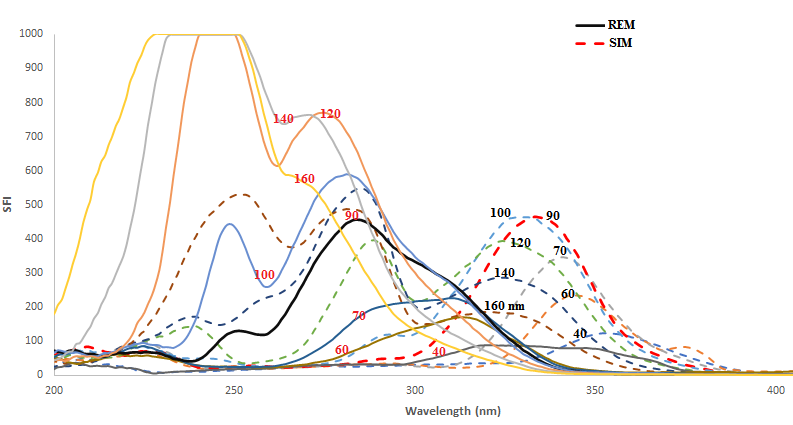


**Supplementary material Fig. S1**


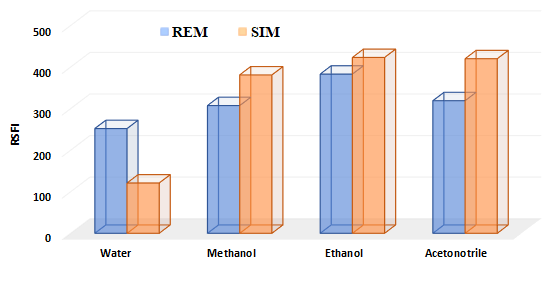


**Supplementary material Fig. S2**

**
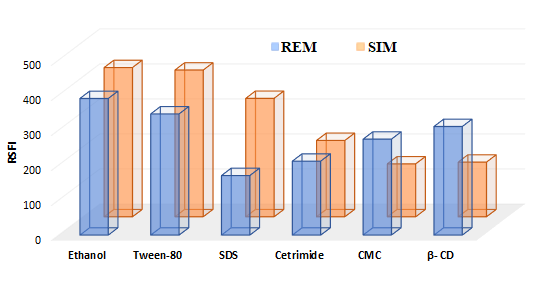
**

**Supplementary material Fig. S3**
